# Acceptance testing of a 0.35 T MR‐Linac: procedures, QA baseline, and system limitations

**DOI:** 10.1002/acm2.70488

**Published:** 2026-02-14

**Authors:** Mateb Al Khalifa, Tianjun Ma, Haya Aljuaid, Siyong Kim, William Y. Song

**Affiliations:** ^1^ Department of Radiation Oncology Virginia Commonwealth University Richmond Virginia USA; ^2^ Department of Radiation Medicine Northwell Health Lake Success New York USA

**Keywords:** acceptance test, MRgRT, MR‐Linac, QA

## Abstract

**Purpose:**

This study describes and evaluates the acceptance procedure for a ViewRay (VR) MRIdian 0.35T MR‐Linac, emphasizing key challenges, limitations, and recommendations to enhance clinical performance and accuracy.

**Methods:**

A comprehensive acceptance test was conducted at a single institution, following the manufacturer's protocols and aligned with established acceptance guidelines. Specific tools and phantoms were used to assess three primary components: mechanical, dosimetric, and Magnetic Resonance Imaging (MRI).

**Results:**

Overall, the test outcomes satisfied the manufacturer's specifications. However, certain issues were identified: high couch attenuation at specific gantry angles (leading to their exclusion from treatment), variations in magnetic field homogeneity at different gantry angles, and discrepancies between TPS calculations and measurements for field output factors smaller than 0.83 cm × 0.83 cm.

**Conclusion:**

This work provides a detailed account of the acceptance testing procedure and establishes a QA baseline for 0.35T MR‐Linac systems. In doing so, it also identifies key system limitations, such as couch attenuation, magnetic field inhomogeneity, and small‐field output discrepancies, underscoring the need for careful gantry angle selection, field homogeneity optimization, and meticulous validation of very small fields.

## 1 INTRODUCTION

Magnetic resonance–guided radiotherapy (MRgRT), delivered using an MR‑Linac, is increasingly being adopted in radiation therapy facilities. By incorporating MRI for gating, MR‑Linac systems offer superior soft‑tissue contrast compared with X‑ray kilovoltage or megavoltage cone‑beam computed tomography (CBCT).^1^ This approach localizes the target and manages motion without additional radiation exposure.^2^, ^3^, ^4^ In addition, MRgRT enables real‑time adaptive planning based on daily anatomical changes.^5^ Furthermore, MRgRT offers potential applications for radiobiological optimization through functional imaging techniques. For example, diffusion‑weighted imaging (DWI) can guide dose painting and adaptive treatment of tumor hypoxia, high‑cellular‑density regions, and local postoperative recurrences.^6^, ^7^, ^8^, ^9^, ^10^

Multiple MRgRT designs, each with different magnetic field strengths and orientations, have been studied.^11^, ^12^, ^13^, ^14^ Currently, three commercial MR‑Linac systems are available: the 0.35 T MR‑Linac (ViewRay, Oakwood, CA, USA), the 0.5 T Aurora RT (MagnetTx Oncology Solutions, Calgary, Alberta, Canada), and the 1.5 T Elekta Unity (Elekta Instrument AB, Stockholm, Sweden).^11^, ^15^, ^16^ This paper focuses on the 0.35 T MR‑Linac, which replaced the original 0.35 T MR‑^60^Co version that has been in clinical use since 2014.^17^, ^18^ The Linac‑based version was introduced in 2017.^19^

Safe and effective dose delivery relies on acceptance testing. Guidelines from AAPM, IPEM, and NCS provide fundamental principles for conventional treatment machines,^20^, ^21^, ^22^, ^23^ but comprehensive acceptance protocols for 0.35T MR‑Linacs remain limited. Because of the unique features of the 0.35 T MR‑Linac, this manuscript outlines acceptance testing procedures to inform quality assurance (QA) efforts.

The aims of this work are threefold: first, to review the acceptance testing framework for the 0.35 T MR‑Linac; second, to establish a baseline for quality assurance; and finally, to highlight observed system limitations encountered during the acceptance phase.

## 2 MATERIALS AND METHODS

### 2.1 0.35T MR‐Linac

#### 2.1.1 System design

The 0.35 T MR‑Linac includes a split superconducting magnet separated by a 28 cm gap, connected both mechanically and thermally to maintain stability. All Linac components occupy this gap so the treatment beam is delivered perpendicularly to the static magnetic field.^19^ A gantry motor rotates the system up to 3 rpm. Six protective shells with ferromagnetic layers shield electronics from magnetic field effects, and additional radiofrequency barriers, including carbon fiber and copper, prevent interference in MR images.^24^ A 70 cm bore serves both MRI and Linac, sharing a single treatment isocenter. A virtual isocenter is located 155 cm from the treatment isocenter. Thus, the MR‑Linac has two isocenters: the treatment isocenter and the virtual isocenter. Laser alignment at the virtual isocenter is critical for linking these two points accurately because patients and phantoms are positioned using the lasers at the virtual isocenter and then moved to the treatment isocenter.

The treatment table moves in vertical, lateral, and longitudinal directions. At the virtual isocenter outside the magnet, vertical travel ranges from 0 mm to −550 mm, and inside the bore it ranges from 0 mm to −200 mm. The table can shift laterally up to ± 7 cm at isocenter, though this range decreases as it lowers. The longitudinal range reaches 2900 mm.

The Linac coordinate system follows the IEC 61217 standard, whereas the MRI employs a DICOM‑based coordinate system. Both systems share a common reference point: the treatment isocenter. The laser system, which also adheres to DICOM conventions, intersects at the virtual isocenter. The coordinate frameworks of all three subsystems are summarized in Table 1.

**TABLE 1:** The coordinate systems used in a 0.35 T MR‐Linac for a head‐first‐supine patient. Each row shows how the conventional anatomical directions (left, superior, and anterior) map onto the Linac (IEC61217), MRI (DICOM), and laser (DICOM) axes.

Head first supine patient	Linac (IEC61217)	MRI (DICOM)	Laser (DICOM)
Left	+X_RT_	+X_MRI_	+X_L_
Superior	+Y_RT_	−Z_MRI_	−Z_L_
Anterior	+Z_RT_	+Y_MRI_	+Y_L_

#### 2.1.2 6 MV FFF Linac

A magnetron generates radiofrequency power for electron acceleration, and the perpendicular magnetic field reduces Faraday‑effect losses. An S‑band waveguide delivers a 6 MV flattening filter free (FFF) beam at 600 cGy per minute, measured at a 90 cm source‑axis distance (SAD). Beam shaping is performed by a double‑stacked, double‑focused high‑resolution MLC with 138 tungsten alloy leaves: 34 pairs in the upper stack and 25 pairs in the lower stack, each leaf 5.5 cm high for a total leaf thickness of 11 cm. A 12 mm vertical gap exists between the stacks. Leaves do not have tongue‑and‑groove edges; instead, the upper and lower stacks are offset by half a leaf width to reduce leakage. Thus, the effective leaf width (MLC resolution) at 90 cm SAD is 4.15 mm (0.415 cm).

#### 2.1.3 0.35T MRI

The 0.35 T magnet is oriented horizontally in a double donut design, providing a gradient strength up to 18 mT/m and a maximum slew rate of 200 T/m/s. Its 80 cm diameter gradient coils include self‑shielding features that reduce noise and stress. Body transmit coils, outfitted with radiofrequency shielding, span the 28 cm gap.^11^ Two surface receiver coils serve each half of the body, featuring a six‑element phased array for the torso and a five‑element phased array for the head and neck. Seven imaging sequences are available: Spin Echo (SE), Gradient Echo (GRE) in 2D and 3D, Turbo Flash (TFL) 3D, Segmented Echo Planar Imaging (EPI) 2D, and True Fast Imaging with Steady‑State Free Precession (TRUFI) in both 2D and 3D.^25^, ^26^ TRUFI is commonly used clinically.

### 2.2 Mechanical

#### 2.2.1 MV isocenter

In compliance with AAPM TG 142,^22^ radiation beams are required to converge at the isocenter, defined as the center of the minimum tangent circle, within a tolerance of ± 1 mm, regardless of beam angle.

As an initial step, a Sun Nuclear IC Profiler™ (ICP) was used to verify laser alignment. The laser system consists of three components: side lasers (left and right) and a sagittal laser. Each laser was aligned and adjusted with the ICP separately. A 5 cm slab of Solid Water® High Equivalency (HE) (Gammex Inc., Wisconsin, USA) was placed behind the ICP on the treatment couch for support. For the sagittal laser, the ICP was placed flat on the couch in its standard horizontal orientation. For the side lasers, the ICP was positioned vertically so that its face was perpendicular to the corresponding laser. At each setup, the ICP was irradiated at the corresponding gantry angle using the maximum field size(FS). The lasers were then adjusted based on the central beam reading recorded by the ICP.

Next, the alignment accuracy of the radiation (RT) and laser (L) isocenters was evaluated using a starshot test, which quantified any shifts in the X(RT,L), Z(RT,L), and Y(RT,L) axes due to gantry rotation. The starshot also determined the size of the radiation isocenter by rotating the gantry through various angles. Because the 0.35 T MR‑Linac design does not permit collimator or couch rotation, the isocenter size was measured solely through gantry rotation; accordingly, the starshot served as both a gantry angle accuracy check and an isocentricity assessment.

**FIGURE 1:**
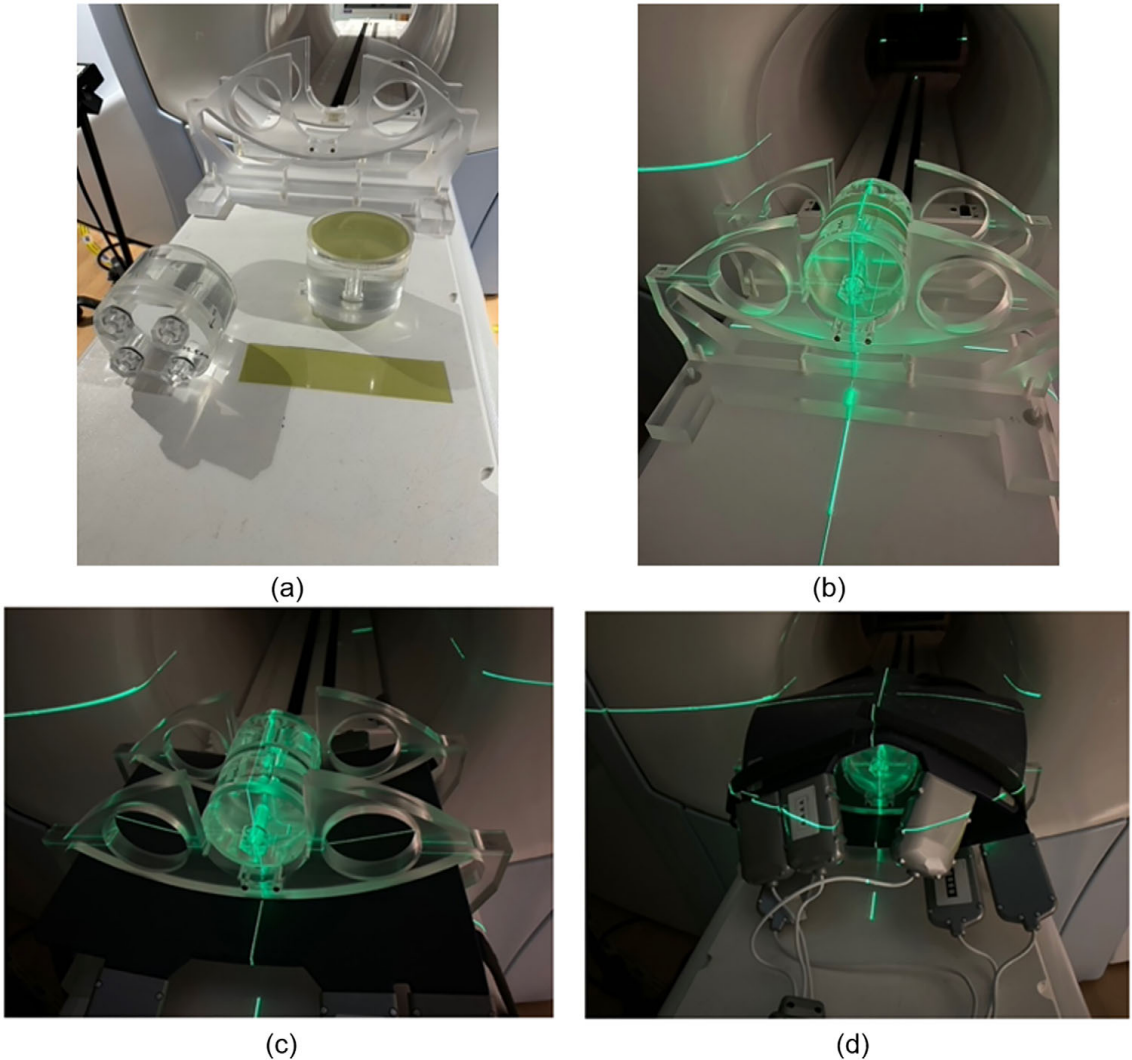
Setup of the VR QA phantom for verification of the radiation and MRI isocenters. Panels (a) and (b) show the configuration used for star‑shot measurements that check coincidence of the radiation (RT) and laser (L) isocenters. (a) The phantom is disassembled to reveal the circular and rectangular EBT3 film inserts. (b) After reassembly, the film‑loaded phantom is aligned with the room lasers at the virtual isocenter before being advanced to the treatment isocenter. Panels (c) and (d) illustrate the arrangement for verifying coincidence of the MRI (MR) and laser isocenters. (c) The phantom is positioned on the lower torso coil and aligned with the lasers at the virtual isocenter. (d) The upper torso coil is added, completing the setup for imaging at the treatment isocenter.

Either a Gafchromic EBT3 film or a Sun Nuclear ArcCheck device may be used for starshot measurements; for this acceptance procedure, EBT3 film was selected. Two pieces of EBT3 film were prepared for the 0.35 T MR‐Linac daily QA phantom (VR QA phantom). One circular piece with a diameter of 13 cm that fits inside the phantom was used to evaluate isocentricity in the X(RT,L) and Z(RT,L) directions, while a rectangular piece measuring 25 × 5 cm was wrapped around the phantom's midsection to assess the Y(RT,L) axis (Figure 1). The EBT3 films were irradiated with 1 × 3 cm^2^ fields at 72° increments of gantry rotation.

#### 2.2.2 Coincidence of laser, radiation and MRI isocenters

The aim of this test was to verify that the MRI and Linac isocenters were aligned within ± 1 mm, in accordance with AAPM TG 142.^22^ The alignment of the Linac (RT) and laser (L) isocenters was addressed in Section 2.1.2. To evaluate the MRI (MR) and laser (L) alignment, the VR QA phantom, equipped with a Torso coil, was aligned to the laser and sent to the treatment isocenter (Figure 1). A TRUFI sequence was then used to measure any shifts of the MRI and laser (L) isocenters (X_L, MRI_, Y_L, MRI_, Z_L, MRI_).

The relative shift of the MR isocenter with respect to the RT isocenter was calculated using the relationship shown in Equation (1):

(1) $$\begin{array}{cc} \left(X_{MR,RT},Y_{MR,RT},Z_{MR,RT}\right) & \equiv \left(X_{MR,L}+X_{L,RT},Y_{MR,L}\right.\\ & \;+\left.Y_{L,RT},Z_{MR,L}+Z_{L,RT}\right) \end{array}$$

#### 2.2.3 MLC radiation FS accuracy

The purpose of this test was to verify the agreement between measured radiation FSs and those computed by the Treatment Planning System (TPS). FS was determined by measuring the Full Width at Half Maximum (FWHM) of beam profiles along the inline and crossline axes. The criterion for passing was a difference of ± 2 mm or less between measured and calculated FSs. The square field side lengths evaluated were 0.83 cm, 1.66 cm, 4.15 cm, 9.96 cm, 14.94 cm, 19.92 cm, and 24.07 cm, corresponding to 0.83 cm × 0.83 cm through 24.07 cm × 24.07 cm. A rectangular field of 27.2 cm × 24.07 cm, corresponding to the maximum FS, was also tested.

For the smaller fields (0.83 cm^2^ and 1.66 cm^2^), Gafchromic EBT3 film was used. Each film was aligned to the laser isocenter with 0.5 cm of solid‐water buildup at the film surface (*SAD*  =  90 cm) and 5 cm of backscatter. FSs of 4.15 cm^2^ or larger were measured using the ICP. The ICP was positioned on the couch surface with its detector array at isocenter, and 0.5 cm of solid water was added on top. Because the ICP has approximately 0.9 cm of inherent buildup, an extra 0.5 cm was needed to achieve the 1.5 cm depth for d_max_ of a 6 MV FFF beam.

The Monte Carlo‐based TPS on the 0.35 T MR‐Linac was used to calculate the FWHM for all FSs. Measured and calculated results were then compared under identical conditions to assess consistency.

#### 2.2.4 MLC transmission and MLC inter‐leaf leakage

The objective of this test, as specified by the 0.35 T MR‐Linac vendor and IEC 60601‐2‐1, was to confirm that the average MLC transmission remains <  0.375% and the maximum MLC transmission, averaged over a 1.0 × 1.0 cm^2^ area, remains <  1%, both relative to a 9.96 × 9.96 cm^2^ reference field at the central axis (CAX) and normalized per MU.

To assess MLC transmission and inter‑leaf leakage, Gafchromic EBT3 film was placed between 5 cm of backscatter and 2 cm of buildup and positioned at 90 cm SAD. A total of 10 000 MU was delivered with the MLC fully closed at gantry angles 0°, 90°, and 270°. For each gantry angle, the film was oriented so that it faced the incident beam.

A reference film was then acquired by delivering a 9.96 × 9.96 cm^2^ open field for 100 MU at a gantry angle of 0° under identical conditions. The EBT3 films were calibrated to cover the expected range of transmission doses. For analysis, a 30 × 30 cm^2^ region of interest (ROI) encompassing the entire leakage field was defined to obtain the average leakage dose, while the maximum leakage dose was obtained by placing a 1.0 × 1.0 cm^2^ ROI centered on the peak leakage region and calculating the mean dose within that 1 cm^2^ area. Both quantities were normalized to the reference field (accounting for the different MU settings) and expressed as percentage transmission using:

(2) $$\%\mathrm{Average}\;\mathrm{Leakage}=\frac{\mathrm{Average}\;\mathrm{Dose}/10\;000\;\mathrm{MU}}{\mathrm{Reference}\;\mathrm{Dose}/100\;\mathrm{MU}}\times 100$$

(3) $$\begin{array}{cc} & \%\mathrm{Maximum}\;\mathrm{Leakage}\\ & \;=\frac{\mathrm{Maximum}\;\mathrm{Dose}/10\;000\;\mathrm{MU}}{\mathrm{Reference}\;\mathrm{Dose}/100\;\mathrm{MU}}\times 100 \end{array}$$

#### 2.2.5 MLC linearity and position accuracy

The 0.35 T MR‐Linac specifications require MLC leaves to maintain linearity within ± 2 mm. To evaluate this linearity and position accuracy, a 13″ × 17″ piece of Gafchromic EBT3 film was used. The film was positioned at the virtual isocenter and secured atop 5 cm of solid‐water backscatter, oriented in landscape relative to the bore axis. An additional 2 cm of solid‐water buildup was placed over the film at *SAD*  =  90 cm, and the entire setup was then moved to the treatment isocenter.

A field measuring 1 × 24.07 cm^2^ was prepared to deliver the irradiation. Strip patterns were created along the Y‐axis (in‐plane) using all MLCs, while a picket fence test was performed at multiple positions along the X‐axis (cross‐plane) at 0, ± 4, ± 8, and ± 12 cm. Each field was delivered with 200 MU. These measurements were repeated at gantry angles of 0°, 90°, 180°, and 270°.

### 2.3 Dosimetric validation

#### 2.3.1 Field profile shape and symmetry

The objective of this test was to evaluate the beam's field profile shape and verify compliance with symmetry requirements. Intensity points along the beam profiles were measured at locations equidistant from the CAX, and the difference between measured values and those calculated by the TPS was required to be within ± 2%. For symmetry, the maximum variation between measured points symmetrically located around the CAX was also constrained to ± 2%.

Measurements were acquired using a calibrated IC Profiler (ICP) with 0.5 cm of solid‑water buildup. The ICP has 0.9 cm of inherent buildup, giving a total buildup of 1.4 cm (at dmax for a 6 MV FFF beam). An additional 5 cm slab of solid‑water was placed behind the ICP to provide backscatter. The ICP was positioned at the CAX, and 200 MU were delivered for each measurement. To facilitate point‑by‑point analysis, profile smoothing was enabled in the IC Profiler software. Data were collected at gantry angles of 0°, 90°, and 270° for two FSs at each angle: 9.96 × 9.96 cm^2^ and 27.2 × 24.07 cm^2^. Off‑axis ratios were measured at ± 2 cm and ± 4 cm for the 9.96 × 9.96 cm^2^ field, and at ± 6 cm and ± 11 cm for the 27.2 × 24.07 cm^2^ field.

Flatness differences were determined by subtracting the measured values from the TPS values, while symmetry differences were calculated by comparing measured points at opposite off‐axis positions relative to the CAX.

#### 2.3.2 Field penumbra

The results for this test were derived from Sections 2.1.4 (MLC radiation FS accuracy) and 2.2.1 (field profile shape and symmetry). Square FSs of 0.83, 1.66, 4.15, 9.96, 14.94, 19.92, and 24.07 cm^2^, as well as 27.2 cm × 24.07 cm, were examined. The penumbra was defined as the distance between the 20% and 80% isodose lines at a depth of 5 cm with an SAD of 90 cm.

For larger fields (≥ 4.15 cm^2^), the beam profiles were normalized before measuring the distance between the 20% and 80% isodose lines. Smaller fields (≤ 1.66 cm^2^) did not require normalization. The measured penumbra was then compared with the TPS values to determine the difference, assessing whether it remained within the ± 1 mm criterion. However, normalizing an FFF profile to 100% yields different penumbra values from those of a conventional flattened beam, so these results cannot be directly compared.

#### 2.3.3 Couch attenuation

The patient couch is constructed from MRI‐safe materials and is designed to support a load of 200 kg. Its treatment surface is made of fiberglass, which minimally attenuates the radiation beam, and these effects are accounted for in the TPS.

This test aimed to measure couch attenuation and verify that the difference between measurements and TPS calculations remained within ± 3%. A VR QA phantom was aligned at the virtual isocenter, and an Exradin^®^ A28MR Ion Chamber was placed inside it and connected to an electrometer at a bias of −300 V. A 9.96 × 9.96 cm field, set to deliver 100 MU, was irradiated at gantry angles of 90°, 140°, 160°, 180°, 200°, 220°, and 270°. Each beam was planned individually for its respective gantry angle.

The averaged readings (R_0_) from beams attenuated at 90° and 270° were then calculated. The relative attenuation at each gantry angle was determined using Equation (4):

(4) $$\mathrm{Relative}\;\mathrm{attenuatio}n_{\mathrm{Meas}}=\left[1-\frac{R_{\theta }}{R_{0}}\right]\times 100$$

where (Rθ) is the measured reading at each gantry angle.

The same procedure was repeated in the TPS under identical conditions; the couch height was matched to the measurement setup, and a dose grid of 0.15 cm was employed. To minimize uncertainty, 1 Gy was prescribed to the isocenter and measured with a small chamber volume (∼0.125 cm^3^). Couch attenuation in the TPS was calculated using Equation (5):

(5) $$\mathrm{Relative}\;\mathrm{attenuatio}n_{\mathrm{Calc}}=\left[1-\frac{\mathrm{MU}}{\mathrm{Reference}}\right]\times 100$$

where MU represents the monitor units required to deliver 1 Gy at the isocenter, and Reference is the average MU needed to deliver 1 Gy at 90° and 270°.

Finally, couch attenuation at each gantry angle was determined by comparing measured and calculated doses.

#### 2.3.4 Reference dosimetry

The reference dosimetry calibration was performed using an MRI‐compatible 1D water tank (PTW, Freiburg, Germany) and a PTW farmer‐type ionization chamber (TN30013) with a valid ADCL calibration. Numerous studies have investigated the impact of the K_B_ factor on the chamber's response in the presence of a magnetic field.^27^, ^28^, ^29^, ^30^, ^31^, ^32^, ^33^ A K_B_ factor of 0.9957 was adopted.^33^ The ion chamber was oriented perpendicular to the magnetic field at a gantry angle of 0°. Measurements were performed at a depth of 10 cm, with %dd(10)x used as the beam quality specifier.

All remaining procedures for the reference dosimetry calibration followed the guidelines of the AAPM Task Group Report 51 addendum.^34^ The calibration was finalized using a setup distance of *SAD* =  90 cm and *SSD* = 80cm.

#### 2.3.5 Field output factor (FOF)

The FOF was measured using an Exradin® W2 (1 mm diameter × 1 mm length) plastic scintillator detector (PSD) (Standard Imaging Inc., USA) to verify agreement with the TPS. All measurements followed IAEA TRS 483 guidelines.^35^ The W2 detector was chosen for its water equivalence and minimal correction‑factor requirements. Data were acquired in a THALES 3D MR SCANNER water tank (LAP GmbH, Germany), using an SSD of 85 cm, an SAD of 90 cm, and a detector depth of 5 cm. The phantom was first aligned at the virtual isocenter and then shifted to the treatment isocenter. Cross‑plane and in‑plane scans were performed to ensure precise detector centering.

The following FSs were tested: 0.2 cm × 0.415 cm, 0.415 × 0.415 cm^2^, 0.83 × 0.83 cm^2^, 1.66 × 1.66 cm^2^, 2.49 × 2.49 cm^2^, 3.32 × 3.32 cm^2^, 6.64 × 6.64 cm^2^, and 8.3 × 8.3 cm^2^, with 9.96 × 9.96 cm^2^ defined as the reference field. Each FS was measured three times and averaged. The FOF was then determined by dividing each field's average reading by that of the reference field. The TPS was also used to compute FOFs for every FS, and these results were compared with measurements to confirm TPS accuracy.

#### 2.3.6 Dose and latency gating

Latency gating tests were performed during the acceptance procedure using a CIRS motion phantom (CIRS, Inc., Norfolk, USA), an A28 MRI‐compatible detector, and a Standard Imaging SuperMAX™ electrometer. The goal was to demonstrate and assess gating latency. Torso coils were placed around the phantom and images were acquired on a 0.35 T MR‑Linac using a clinical TRUFI sequence. A conformal treatment plan was then generated in the 0.35 T MR‑Linac TPS. A single field at gantry angle 0° was created, with the aperture conformed to the gating target. A large number of MUs was prescribed to ensure a sufficient number of gating events during delivery, enabling statistical analysis of beam‑on and beam‑off times for latency determination. For phantom irradiation, the planned setup was reproduced, with cine images acquired in the sagittal plane, applying a gating margin of 3 mm in the longitudinal direction, along which the target entered and exited the irradiation field, and 5 mm in the remaining directions. The programmed sinusoidal waveform had an amplitude of 12 mm and a period of 4 s. Beam‑on and beam‑off gating latency was verified by analyzing the beam‑control signal and the corresponding change in beam status.

Electrometer charge readings were taken for both gating and non‑gating deliveries, and the percent deviation was calculated as

(6) $$Deviation\;\left(\%\right)=100\times \frac{\left(D_{ng}-D_{g}\right)}{D_{ng}}$$

where D_ng_ is the charge for non‑gating delivery and D_g_ is the charge for gating delivery.

### 2.4 MRI verification

#### 2.4.1 B_0_ field homogeneity

Magnetic field homogeneity was evaluated by assessing deviations in the central frequency ƒ*_0_* = 14.70176 ± 0.015 MHz, corresponding to a permissible range of 14.68676 MHz–14.71676 MHz. The 0.35 T MR‐Linac manufacturer recommends a peak‐to‐peak field inhomogeneity below 5 ppm over a 24 cm diameter spherical volume (DSV), indicating that the system remains within acceptable limits.

**FIGURE 2:**
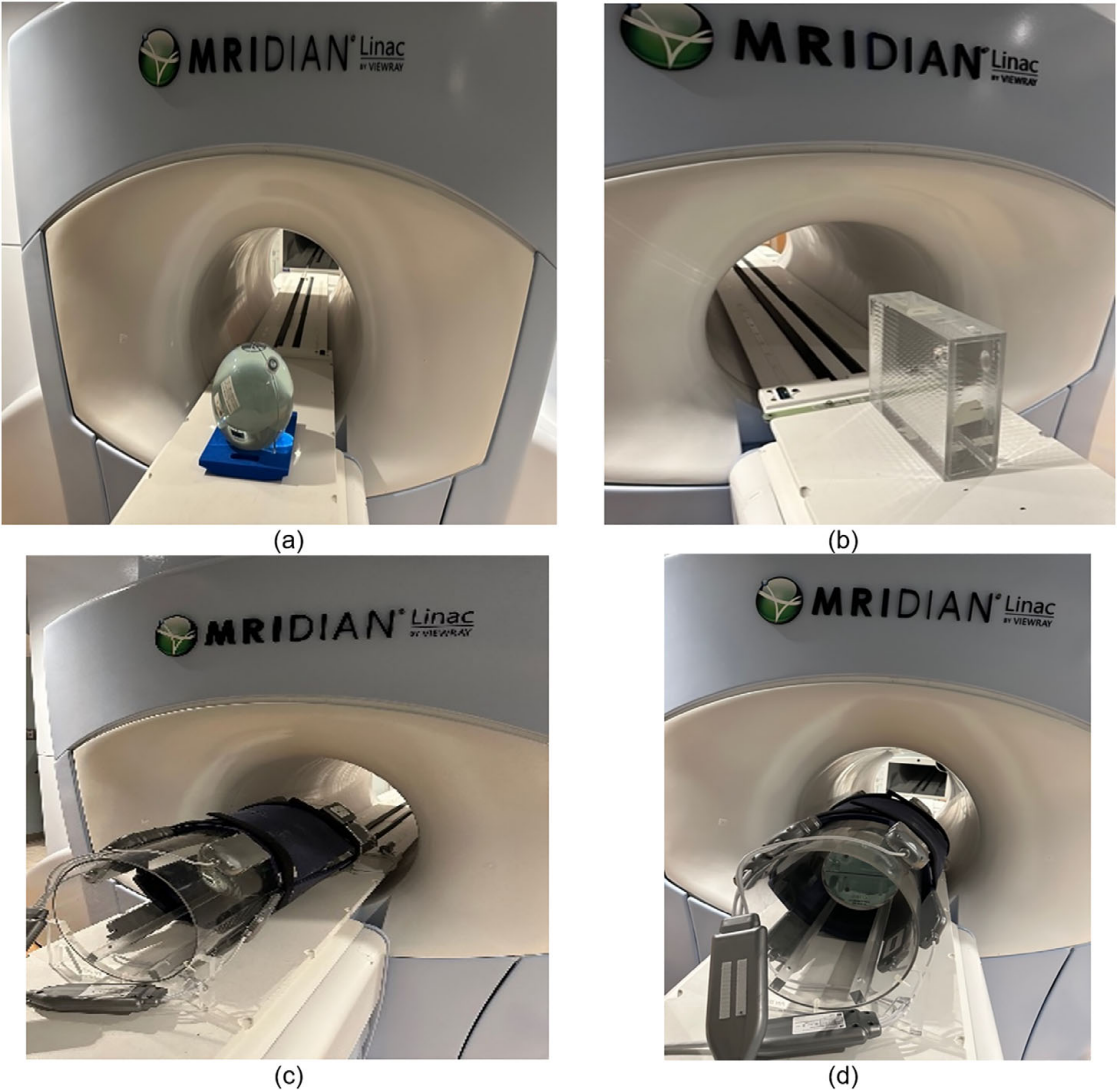
MRI phantoms used for acceptance testing. (a) A 24 cm spherical phantom is positioned and then moved to the treatment isocenter to assess B0 homogeneity. (b) The spatial integrity phantom is centered on the couch and subsequently moved to the MRI isocenter. (c) The same spherical phantom is placed inside the torso coils and transferred to the MRI isocenter for SNR and uniformity measurements. (d) The ACR body phantom is loaded in the torso coils and moved to the MRI isocenter.

This homogeneity test involved placing a 24 cm spherical phantom at the MRI isocenter (Figure 2a). A ppm check protocol, provided by the 0.35 T MR‐Linac manufacturer, used TR/TE = 3000/0.35 ms, a 90° flip angle, and a receive bandwidth (rBW) of 1260 Hz/Px. Acquisitions were made at 30° increments covering the entire range of gantry angles, and the raw data for each angle were saved. Worst‐case homogeneity corresponded to the largest FWHM, while best‐case homogeneity corresponded to the smallest FWHM.

#### 2.4.2 Spatial integrity

This test evaluated the MRI system's ability to produce images with a spatial accuracy of less than 2 mm for spherical volumes ranging from 20 to 35 cm in diameter within the field of view (FOV).^36^ It also specified achieving an accuracy of less than 1 mm for spheres measuring 20 cm in diameter within the FOV.

To acquire images in axial, sagittal, and coronal orientations, a Spatial Integrity (SI) phantom measuring 330 × 330 × 100 mm was used. Scans were performed at the CAX for the axial, coronal, and sagittal planes. In the sagittal plane, off‐axis orientations of ± 7 cm and ± 12.5 cm were included to encompass the full imaging FOV (Figure 2b).

Three MRI sequence protocols were provided by the 0.35 T MR‐Linac manufacturer to assess spatial integrity. The TRUFI protocol used TR/TE  =  3.36/1.44 ms, a flip angle of 60°, an FOV of 350 × 350 × 350 mm^3^, an imaging matrix of 234 × 234, a voxel size of 1.5 × 1.5 × 3 mm^3^, and a receive bandwidth (rBW) of 534 Hz/Px. The True SE protocol used TR/TE  =  1200/109 ms, a flip angle of 180°, an FOV of 350 × 350 × 350 mm^3^, an imaging matrix of 234 × 234, a voxel size of 1.5 × 1.5 × 3 mm^3^, and an rBW of 70 Hz/Px. Finally, the TrueFlash protocol used TR/TE  =  1130/2.75 ms, a flip angle of 15°, an FOV of 350 × 350 × 350 mm^3^, an imaging matrix of 234 × 234, a voxel size of 1.5 × 1.5 × 3 mm^3^, and an rBW of 150 Hz/Px.

#### 2.4.3 SNR and uniformity

The objective of this test was to verify the integrity of the MRI coils by evaluating their Signal‐to‐Noise Ratio (SNR) and uniformity. NEMA methods were applied to assess SNR and uniformity for the body, torso, and head/neck coils. A 24 cm spherical phantom was positioned at the virtual isocenter for each coil test and then moved to the treatment isocenter. Figure 2c illustrates a representative setup using this phantom for torso coil assessment. A localizer acquisition was performed to confirm the phantom's position.

The manufacturer provides NEMA protocols for each coil in transverse, sagittal, and coronal orientations. These protocols specify: TR/TE  =  1500/15 ms, flip angle  =  90°, FOV  =  300 × 300 × 350 mm^3^, imaging matrix  =  256 × 256, voxel size  =  1.2 × 1.2 × 10 mm^3^, and rBW  =  130 Hz/Px. The phase‐encoding direction for axial and sagittal scans is anterior–posterior, while for coronal scans it is right–left.

The acceptance criterion for SNR was ≥ 12 for the body coil, and >  30 (sagittal and transverse) or >  25 (coronal) for the torso and head/neck coils. The SNR was calculated using Equation (6):

(7) $$\mathrm{SNR}=\left[\frac{0.66\times \mathrm{ROI}\;\mathrm{signal}\;\mathrm{mean}}{\mathrm{ROI}\;\mathrm{Noise}\;\mathrm{SD}}\right]$$

Meanwhile, the uniformity, calculated using Equation (7), was required to be ≥ 60 % for the body coil and >  50 % for the torso and head/neck coils:

(8) $$\begin{array}{cc} & \mathrm{Uniformity}\;\%\\ & \;=100\times \left(1-\left[\frac{\mathrm{ROI}\;\mathrm{Signa}l_{\max}-\mathrm{ROI}\;\mathrm{Signa}l_{\min}}{\mathrm{ROI}\;\mathrm{Signa}l_{\max}+\mathrm{ROI}\;\mathrm{Signa}l_{\min}}\right]\right) \end{array}$$

#### 2.4.4 Image quality

The American College of Radiology (ACR) body phantom was used to perform image quality acceptance tests, evaluating percent signal ghosting, percent integral uniformity, low‐ and high‐contrast visibility, slice thickness, and position accuracy (Figure 2d). The 0.35 T MR‐Linac manufacturer provided two MRI scanning protocols for 2D T1‐ and T2‐weighted imaging. The T1‐weighted protocol was configured with TR/TE  =  500/20 ms, a flip angle of 90°, an FOV of 250 × 250 × 350 mm^3^, an imaging matrix of 256 × 256, a voxel size of 1 × 1 × 5 mm^3^, and rBW  =  78 Hz/Px. The T2‐weighted protocol was configured with TR/TE  =  2000/80 ms, a flip angle of 90°, an FOV of 250 × 250 × 350 mm^3^, an imaging matrix of 256 × 256, a voxel size of 1 × 1 × 5 mm^3^, and rBW  =  78 Hz/Px. Images were subsequently analyzed manually.

## 3 Results

### 3.1 Mechanical

#### 3.1.1 MV isocenter

**FIGURE 3:**
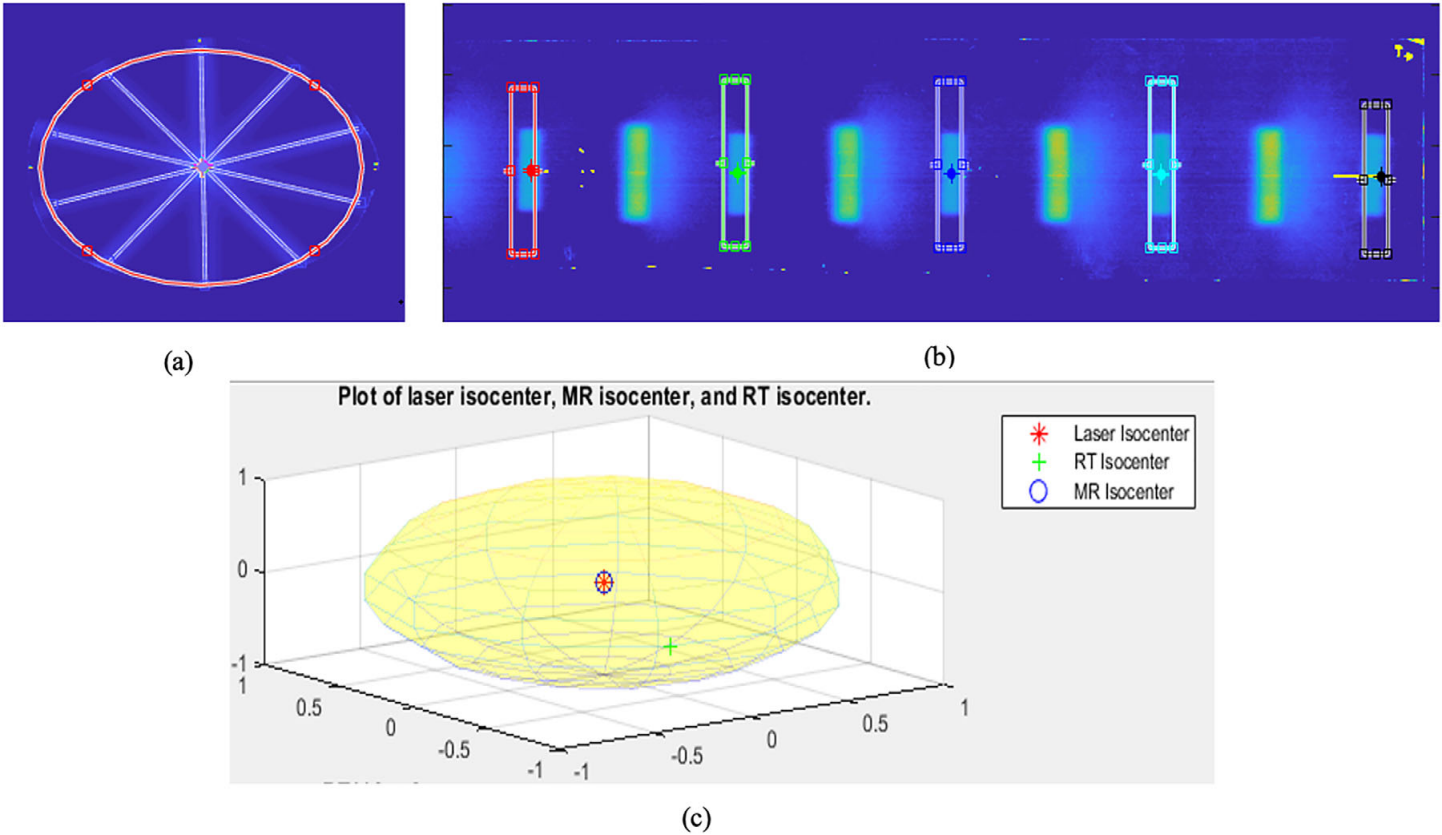
Starshot film results for evaluating the MV isocenter size. (a) Representation of the lateral (X_RT,L_) and vertical (Z_RT,L_) axes. (b) Illustration of the longitudinal (Y_RT,L_) axis. (c) A 3D plot showing the coincidence of the laser, radiation, and MRI isocenters. Note that the laser isocenter is defined at the virtual isocenter, and the treatment isocenter's accuracy is assessed relative to this reference. “RT” denotes the radiation isocenter and “MR” denotes the MRI isocenter, both located at the treatment isocenter.

The laser alignment was adjusted to center at the virtual isocenter within a tolerance of ≤ 1 mm. ICP reading profiles for each laser side verified that all lasers converged at the virtual isocenter and, consequently, at the treatment isocenter. Figure 3 shows the EBT3 starshot test results, where the isocenter has a radius of 0.6 mm.

#### 3.1.2 Coincidence of laser, radiation and MRI isocenters

The MRI‑to‑laser shifts were zero because the laser was first aligned to the MRI images. By contrast, the radiation (RT)‑to‑laser shifts measured 0.0 mm laterally, −0.4 mm longitudinally, and −0.5 mm vertically. Therefore, the inferred MRI‑to‑radiation (MR‑RT) shifts were 0.0 mm, +0.4 mm, and +0.5 mm in the lateral, longitudinal, and vertical directions, respectively.

#### 3.1.3 MLC radiation FS accuracy

**FIGURE 4:**
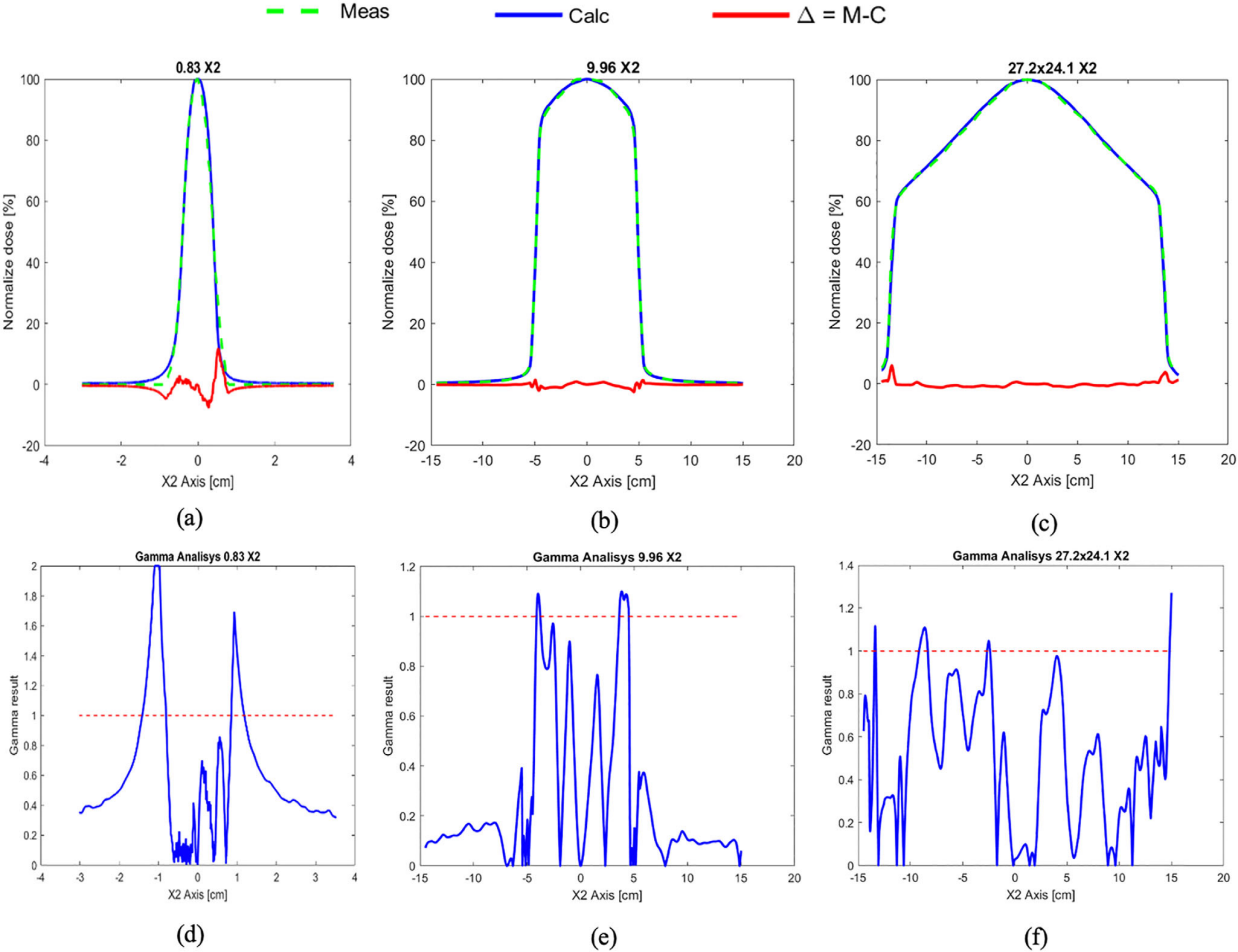
Representative MLC radiation field discrepancies between TPS calculations and measurements in the cross‐plane (X) direction. The top row (a–c) shows profile plots for FSs of 0.83 cm × 0.83 cm, 9.96 cm × 9.96 cm, and 27.2 cm × 24.07 cm, respectively, while the bottom row (d–f) depicts the corresponding gamma analyses for each FS.

Table 2 summarizes the discrepancies between measured and calculated FSs. The greatest deviation in the cross‐plane direction (0.1 cm) was observed for a 27.2 cm × 24.02 cm field, whereas the largest in‐plane discrepancy (0.13 cm) occurred for a 19.92 cm^2^ field. Figure 4 presents representative profile comparisons and the corresponding gamma analyses, using dose‑difference and distance‑to‑agreement criteria of 3 % and 3 mm, respectively.

**TABLE 2:** MLC radiation field accuracy, showing differences between TPS‐calculated and measured FSs in both the cross‐plane and in‐plane directions.

	Cross‐plane (cm)			In‐plane (cm)		
Field size (cm^2^)	TPS	Measured	Difference	TPS	Measured	Difference
0.83	0.79	0.81	0.01	0.88	0.89	0.02
1.66	1.57	1.57	0.00	1.69	1.73	0.03
4.15	4.11	4.14	0.03	4.22	4.23	0.02
9.96	9.84	9.90	0.06	10.08	10.12	0.05
14.94	14.89	14.87	−0.01	15.13	15.22	0.09
19.92	19.88	19.94	0.06	20.16	20.29	0.13
24.07	24.08	24.14	0.06	24.26	24.28	0.03
27.2 × 24.07	27.21	27.31	0.10	24.26	24.29	0.03

[Tolerance: ± 0.2 cm].

#### 3.1.4 MLC transmission and inter‐leaf leakage

Table 3 shows average and maximum MLC leakage percentages at gantry angles of 0°, 90°, and 270°. The average leakage at these angles was 0.08%, 0.03%, and 0.05%, respectively, while the maximum leakage was 0.36%, 0.19%, and 0.23%. Notably, the highest average and maximum leakage levels both occurred at 0°.

**TABLE 3:** Average and maximum MLC leakage percentages at gantry angles of 0°, 90°, and 270°, referenced to a 9.96 cm × 9.96 cm field.

Gantry angle	MLC configuration	MU	Avg MLC leakage %	Max MLC leakage %
0	9.96 cm × 9.96 cm (reference)	100	1.00	1.00
0	Upper/Lower stack ± 0.2 cm	10000	0.08	0.36
90		10000	0.03	0.19
270		10000	0.05	0.23

[Tolerance: maximum MLC transmission < 1%, average MLC transmission < 0.375%].

#### 3.1.5 MLC linearity and position accuracy

Leaf position deviations from the expected centerline remained within the ± 2 mm tolerance. The mean (± SD) peak positions were 0.03  ±  0.28, 0.10  ±  0.28, 0.03  ±  0.28, and 0.01  ±  0.24 mm for gantry angles of 0°, 90°, 180°, and 270°, respectively. Table 4 details the positions of all MLCs at each peak location, along with the average shift per gantry angle.

**TABLE 4:** Position and average shift measurements for peak positions at 0, ± 4, ± 8, and ± 12 cm in the cross‐plane direction for a 1 cm × 24.07 cm field, measured at gantry angles of 0°, 90°, 180°, and 270°. All values are in centimeters.

Plan peak positions in the cross plane direction	Gantry angles							
	0° degree		90° degree		180° degree		270° degree	
	Position	Shift	Position	Shift	Position	Shift	Position	Shift
−12	−11.97	0.03	−11.98	0.02	−11.99	0.01	−11.97	0.03
−8	−8.00	0.00	−7.99	0.01	−7.99	0.01	−7.99	0.01
−4	−4.05	−0.05	−4.04	−0.04	−4.05	−0.05	−4.04	−0.04
0	0.00	0.00	0.00	0.00	0.00	0.00	0.00	0.00
4	4.03	0.03	4.03	0.03	4.03	0.03	4.02	0.02
8	7.99	−0.01	8.00	0.00	7.99	−0.01	7.98	−0.02
12	12.02	0.02	12.05	0.05	12.03	0.03	12.01	0.01

[Tolerance: ± 0.2 cm].

### 3.2 Dosimetric validation

#### 3.2.1 Field profile shape and symmetry

All measured field profile shapes and symmetry results satisfied the acceptance criteria. Symmetry was evaluated at gantry angles of 0°, 90°, and 270° for fields measuring 9.96 × 9.96 cm^2^ and 27.2 × 24.07 cm^2^. Table 5 presents the in‐plane and cross‐plane symmetry for off‐axis points of ± 2 cm and ± 4 cm (9.96 cm × 9.96 cm) and ± 6 cm and ± 11 cm (27.2 cm × 24.07 cm) under each respective gantry angle.

**TABLE 5:** Field‑profile flatness and symmetry at gantry angles of 0°, 90°, and 270° for FSs of 9.96 × 9.96 cm^2^ and 27.2 × 24.07 cm^2^. At each off‑axis point (OAP), the table lists the calculated value from the TPS, the measured value from the IC Profiler (M), the pointwise flatness difference F (%) = M–TPS, and the symmetry S (%) computed from the measured values at ± OAP. X‑profile denotes the cross‑plane direction; Y‑profile denotes the in‑plane direction. Off‑axis points were ± 2 cm and ± 4 cm for the 9.96 × 9.96 cm^2^ field, and ± 6 cm and ± 11 cm for the 27.2 × 24.07 cm^2^ field.

			X profile				Y profile			
G (deg)	FS (cm^2^)	OAP (cm)	TPS (%)	M (%)	F (%)	S (%)	TPS (%)	M (%)	F (%)	S (%)
0	9.96 × 9.96	−2	97.82	97.49	−0.33	−0.15	97.94	97.78	−0.16	0.04
		2	97.85	97.64	−0.21		97.9	97.74	−0.16	
		−4	92.32	91.48	−0.84	−0.27	92.46	92.19	−0.27	0.07
		4	92.36	91.75	−0.61		92.37	92.12	−0.25	
	27.2 × 24.07	−6	85.38	84.44	−0.94	−0.38	85.47	85.12	−0.35	0.11
		6	85.43	84.82	−0.61		85.36	85.01	−0.35	
		−11	68.22	67.85	−0.37	−0.55	68.22	68.49	0.27	0.15
		11	68.3	68.4	0.1		68.06	68.34	0.28	
90	9.96 × 9.96	−2	97.82	98.02	0.2	0.15	97.94	98	0.06	−0.11
		2	97.85	97.87	0.02		97.9	98.11	0.21	
		−4	92.32	92.55	0.23	0.28	92.46	92.35	−0.11	−0.2
		4	92.36	92.27	−0.09		92.37	92.55	0.18	
	27.2 × 24.07	−6	85.38	85.58	0.2	0.39	85.47	85.34	−0.13	−0.28
		6	85.43	85.19	−0.24		85.36	85.62	0.26	
		−11	68.22	69.05	0.83	0.57	68.22	67.74	−0.48	−0.41
		11	68.3	68.48	0.18		68.06	68.15	0.09	
270	9.96 × 9.96	−2	97.82	97.7	−0.12	−0.37	97.94	97.88	−0.06	−0.03
		2	97.85	98.07	0.22		97.9	97.91	0.01	
		−4	92.32	91.98	−0.34	−0.69	92.46	92.18	−0.28	−0.06
		4	92.36	92.67	0.31		92.37	92.24	−0.13	
	27.2 × 24.07	−6	85.38	84.83	−0.55	−0.96	85.47	85.25	−0.22	−0.1
		6	85.43	85.79	0.36		85.36	85.35	−0.01	
		−11	68.22	68.02	−0.2	−1.42	68.22	67.64	−0.58	−0.14
		11	68.3	69.44	1.14		68.06	67.78	−0.28	

[Tolerance: ± 2 %. Abbreviations: G, gantry angle (deg); FS, field size (cm^2^); OAP, off‑axis point (cm); TPS, treatment planning system (calculated) values; M, measured values (IC Profiler); F, flatness, pointwise difference (M–TPS, %); S, symmetry from measured profiles (*S* = M(−OAP)–M(+OAP), %). Symmetry is reported once per ± OAP pair].

#### 3.2.2 Field penumbra

Table 6 presents both measured and calculated penumbra sizes for field dimensions of 0.83, 1.66, 4.15, 9.96, 14.94, 19.92, 24.07 cm^2^, and 27.2 cm × 24.07 cm. All differences remained within the ± 1 mm tolerance. The largest discrepancy was observed for the 4.15 cm field, where the cross‐plane and in‐plane penumbra measured 0.8 mm and 0.7 mm, respectively.

**TABLE 6:** Penumbra discrepancies (in millimeters) for various FSs, comparing measured and TPS‐calculated values in both the cross‐plane and in‐plane directions.

Field size (cm^2^)	Cross‐plane penumbra (mm)			In‐plane penumbra (mm)		
	Measured	TPS	Difference	Measured	TPS	Difference
0.83	3.1	2.5	0.5	3.1	2.7	0.4
1.66	3.5	3.1	0.5	4.0	3.6	0.4
4.15	6.3	5.6	0.8	5.8	5.2	0.7
9.96	7.8	7.3	0.5	7.9	7.5	0.4
14.94	6.3	6.2	0.0	5.7	6.0	−0.2
19.92	6.4	6.3	0.0	5.5	5.9	−0.4
24.07	6.0	6.1	−0.1	5.3	5.4	−0.1
27.2 × 24.07	5.3	5.7	−0.4	5.3	5.4	−0.1

[Tolerance: ≤ 1 mm].

#### 3.2.3 Couch attenuation

Across all gantry angle plans, the maximum deviation remained within ± 1%, with the most pronounced discrepancy observed at 180° between TPS and measurement.

Table 7 summarizes the relative attenuation values from both measurement and TPS calculations, alongside the corresponding deviations at each gantry angle. Notably, the highest relative couch attenuation occurred at 140° and 220° in both the measured and calculated results.

**TABLE 7:** Comparison of measured and TPS‐calculated couch attenuation at various gantry angles. The table shows the relative attenuation in both measurement and TPS, along with the percent deviation between the two.

	Measurement	TPS	
Gantry angle	Rel. attenuation	Rel. attenuation	Deviation (%)
90	1.001	1	0.1
140	1.195	1.197	−0.2
160	1.152	1.159	−0.6
180	1.144	1.155	−1.0
200	1.157	1.159	−0.2
220	1.201	1.197	0.3
270	0.998	1	−0.2

[Tolerance: ± 1 %].

#### 3.2.4 Reference dosimetry

The beam quality specifier %dd(10)x was measured as 64%. In accordance with the AAPM TG‑51 addendum, the k_Q_ value for %dd(10)x was determined by interpolating between 63% and 66%, yielding *k_Q_*  =  0.9947. The dose to water at d_max_ was specified at 1.004 cGy/MU.

3.2.5

Table 8 lists the W2‐based FOF measurements. For FSs of 1.66 × 1.66 cm^2^, 2.49 × 2.49 cm^2^, 3.32 × 3.32 cm^2^, 6.64 × 6.64 cm^2^, and 8.3 × 8.3 cm^2^, the measured values agreed with the TPS calculations within 1%. However, for the 0.83 × 0.83 cm^2^ field, the discrepancy reached −2.3%. More substantial deviations occurred for the smallest fields, 0.2 cm × 0.415 cm and 0.415 × 0.415 cm^2^, with differences of −23.2% and −11.9%, respectively.

**TABLE 8:** FOF measurements at selected FSs. Values obtained with the W2 PSD are compared with those calculated by the TPS, and the percent difference between the two is also reported.

FS (cm^2^)	Measured FOF (W2 PSD)	TPS FOF	Difference (%)
0.2 × 0.415	0.151	0.197	−23.2%
0.415	0.444	0.504	−11.9%
0.83	0.686	0.702	−2.3%
1.66	0.845	0.841	0.4%
2.49	0.889	0.889	0.0%
3.32	0.912	0.911	0.1%
6.64	0.970	0.970	0.0%
8.3	0.986	0.986	0.0%
9.96	1	1	0.0%

[Tolerance: Not specified by the manufacturer].

#### 3.2.6 Dose and latency gating

The beam‐off latency was approximately 100 ms at 4 fps and 90 ms at 8 fps. During non‑gated delivery, the recorded charge was −11.76 nC, compared with −11.77 nC for gated delivery. This difference corresponded to a 0.09% deviation between the two delivery patterns.

### 3.3 MRI verification

#### 3.3.1 B_0_ field homogeneity

**FIGURE 5:**
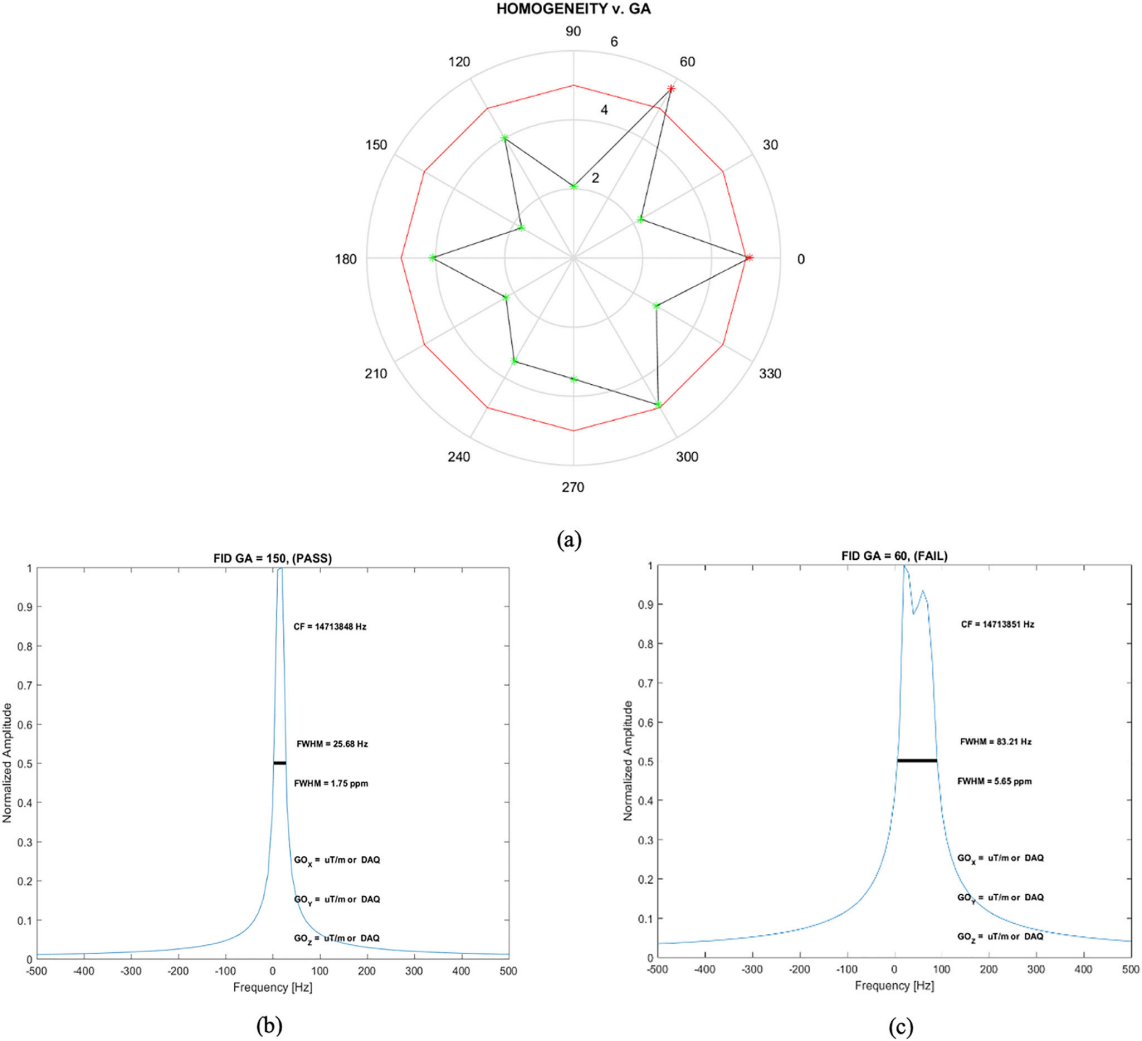
Variation in magnetic field homogeneity at discrete gantry angles. (a) Radar plot of magnetic homogeneity values (green stars, in ppm). (b) Frequency profiles at 14,713,851 Hz, demonstrating optimal homogeneity at a gantry angle of 150°, with an FWHM of 25.68 Hz (1.75 ppm). (c) The least favorable homogeneity occurs at 60°, where the FWHM is 83.21 Hz (5.65 ppm), exceeding the 5 ppm tolerance. CF stands for central frequency, and the FWHM in ppm is obtained by dividing the FWHM (in Hz) by the CF. GO denotes gradient offset in the X, Y, and Z directions, and DAQ stands for data acquisition.

FWHM values (in ppm) derived from the frequency profile (Hz) were used to evaluate magnetic homogeneity. For gantry angles of 0°, 30°, 60°, 90°, 120°, 150°, 180°, 210°, 240°, 270°, 300°, and 330°, the FWHM values were 5.1, 2.2, 5.7, 2.1, 4.0, 1.7, 4.1, 2.3, 3.4, 3.5, 4.9, and 2.8 ppm, respectively. The lowest FWHM (1.7 ppm) at 150° indicated optimal homogeneity, while the highest (5.7 ppm) at 60° indicated the least favorable. Figure 5 provides a radar plot of these values and representative frequency profiles for the best and worst homogeneity conditions.

#### 3.3.2 Spatial integrity

All scan orientations met the spatial‐integrity criteria within the SI phantom's analysis region. With a 20 cm‐diameter field of view, the measured spatial deviations were 0.50 mm, 0.49 mm, and 0.53 mm for the axial, sagittal, and coronal orientations, respectively, using the TRUFI sequence; 0.43 mm, 0.55 mm, and 0.48 mm with TFL; and 0.96 mm, 0.52 mm, and 0.40 mm with TSE.

#### 3.3.3 SNR and uniformity

All scanning orientations met the specified SNR and uniformity requirements for the body, head/neck, and torso coils. Body coil SNRs were 14.8, 14.8, and 14.7 for the axial, sagittal, and coronal orientations, respectively; corresponding values were 61.6, 57.3, and 45.5 for the head and neck coil and 57.3, 51.3, and 43.6 for the torso coil. Uniformity measured 73 , 69 , and 68 for the body coil; 89, 86, and 86 for the head and neck coil; and 90, 87, and 87 for the torso coil in the axial, sagittal, and coronal planes, respectively.

#### 3.3.4 Image quality

The test outcomes for both 2D T1‐weighted and T2‐weighted images are summarized in Table 9.

**TABLE 9:** ACR body phantom results for 2D T1‐ and T2‐weighted imaging.

	Slice number	Measured T1	Measured T2	Tolerance
Percent signal ghosting	7	0.01335	0.00104	< 0.025
Percent integral uniformity (%)	7	97.6	97.4	> 87.5%
Total low‐contrast object detectability	From: 8 to 11	30	22	> 18
High contrast resolution resolving (mm)	1	1	1	≥ 0.9 mm
Slice thickness accuracy (mm)	1	5.57	5.52	5.0 ± 0.7 mm
Slice position accuracy (mm)	1 & 11	0.55	0.5	≤ 5 mm

## 4 DISCUSSION

In this study, we performed acceptance tests on the 0.35T MR‐Linac, focusing on three main components: mechanical, dosimetric validation, and MRI verification. Table S1, located in the appendix as supplementary material, summarizes the entire acceptance‑test process, outlining each test category, its criteria, the phantoms and devices used, and general comments.

Several studies have examined acceptance testing from various perspectives. Ahtesham et al. investigated the 0.35T MR component of the 0.35T MR‐Linac system at twelve different institutions.^37^ They quantified the variability in B_0_ field homogeneity, SNR/uniformity, and ACR image quality testing, as well as spatial integrity, using the manufacturer's acceptance‐test equipment. They observed a strong gantry‐angle dependence for the B_0_ field homogeneity, with median values below 4 ppm for all gantry angles. They also reported that SNR/uniformity, ACR image quality, and spatial integrity met the acceptance criteria. However, their study did not address the mechanical or dosimetric aspects of the acceptance process.

In another study, Shanti et al. focused on the MR components of the 0.35T MR‐Linac using a large MR QA phantom.^38^ They employed three phantoms—a 0.35T MR‐Linac cylindrical water phantom, a Fluke 76–907 uniformity/linearity phantom, and a Modus QA large FOV MRgRT Insight phantom—imaging them in a single scan mode (TRUFI sequence). Their investigations covered a FOV up to 400 mm, simulating a human torso. They observed a higher geometric distortion of 0.8  ±  0.4 mm in the peripheral region (300–400 mm) of the Insight phantom. Their work highlighted the benefits of a multifunctional large FOV phantom for more extensive MR image‐quality tracking compared to the default daily and monthly QA phantoms provided by the manufacturer. However, they did not include the mechanical or dosimetric validation components of the acceptance process and focused solely on the 0.35T MR mode, recommending a single large FOV phantom.

Valdenaire et al. discussed the acceptance, and periodic quality assurance of the 0.35T MR‐Linac.^39^ They briefly addressed acceptance tests for image quality, isocenter position, gantry angles, and MLC positioning using film dosimetry. Their work also covered beam profiles, depth dose, and output factors, measured with PTW microDiamond and PTW SemiFlex detectors, which they compared to Monte Carlo‐based 0.35T MR‐Linac TPS calculations. They found that image quality and MLC positioning were within tolerances, except for the coincidence of radiation, MRI, and laser isocenters (1.4  ±  0.3 mm diameter), which exceeded the 1 mm specification. In addition, they used microDiamond and SemiFlex detectors without applying correction factors for a 0.35T field, introducing potential uncertainties. Their FOF measurements were also limited to a minimum FS of 0.415 cm and did not explore the smallest 0.2 cm × 0.415 cm field achievable by the 0.35T MR‐Linac.

In the present study, we reported all aspects of the 0.35T MR‐Linac acceptance test, including mechanical, dosimetric, and MRI components. The mechanical tests addressed coincidence of radiation, MRI, and laser isocenters; MLC parameters (field‐size accuracy, transmission, interleaf leakage, linearity, and positional accuracy). The dosimetric tests covered field‐size metrics (profile shape, symmetry, penumbra), couch attenuation, output constancy versus gantry angle, reference dosimetry, FOF, and dose/latency gating. For the MRI component, we verified magnet field homogeneity, spatial integrity, SNR, uniformity, and ACR image‐quality criteria.

A few additional considerations arose during acceptance testing, which can guide ongoing QA. First, the system does not include an Optical Distance Indicator (ODI) or a radiation field light, so ensuring accurate calibration of the lasers at the virtual isocenter (155 cm from the treatment isocenter) is crucial for locating all isocenters within ± 1 mm. Because the 0.35T MR‐Linac has no jaws and uses MLCs without tongue‐and‐groove edges, routine checks for MLC positioning, transmission, and interleaf leakage remain particularly important. We also found that couch angles around 140° and 220° incur high attenuation in both measurements and TPS calculations and are therefore best avoided in clinical treatments.

**FIGURE 6:**
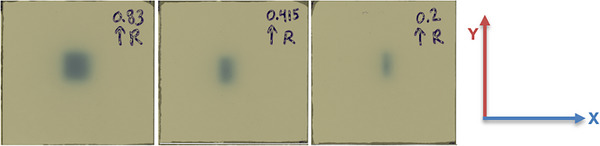
Irradiated EBT3 film scans illustrating unexpected MLC shaping for the smallest fields. FSs presented: (a) 0.83 cm × 0.83 cm, (b) 0.415 cm × 0.415 cm, and (c) 0.2 cm × 0.415 cm. Panel (d) shows the coordinate system, where the Y‑axis represents the longitudinal (in‑plane) direction and the X‑axis represents the lateral (cross‑plane) direction. The handwritten numbers denote the nominal FSs, the arrows indicate the gantry direction relative to the film, and the letter “R” marks the right side for orientation. For the smallest fields, (b) and (c), the in‑plane dimension is not shaped as nominally planned and is, in practice, enlarged to approximately 0.83 cm. This effect is observed for FSs less than 0.83 cm.

For FOF measurements, we used an Exradin^®^ W2 PSD (requiring a correction factor of 1 under TRS 483)^35^ and scaled the readings from a 9.96 cm × 9.96 cm reference field down to 0.2 cm × 0.415 cm. We observed notable discrepancies between TPS calculations and these measurements beginning at 0.83 cm × 0.83 cm and continuing through the smallest fields: the 0.2 cm × 0.415 cm field exhibited a −23.2% deviation, while the 0.415 cm × 0.415 cm field showed −11.9% (Table 8). Film measurements confirmed that these smallest fields are not shaped as nominally planned and are, in practice, larger than expected (Figure 6). Specifically, the measured FS for the nominal 0.2 cm × 0.415 cm field was 0.34 cm × 0.80 cm, and for the 0.415 cm × 0.415 cm field it was 0.48 cm × 0.85 cm. This inconsistency may stem from the MLC upper/lower stack specification (±  0.2 cm), which can introduce inaccuracies in leaf positioning for very small fields. Furthermore, Ahtesham et al.^40^ conducted a study on the 0.35 T MRI‐Linac that involved a multi‐institutional comparison of dosimetric data. They found that the FOF results for FSs ≤  0.83 cm × 0.83 cm exhibited large variations. Further investigations are recommended to assess MLC positioning, TPS modeling, and the clinical feasibility of fields below 0.83 cm × 0.83 cm. In the interim, we recommend using less demanding plan fluences to reduce the likelihood of extremely high‑MU segments.

**FIGURE 7:**
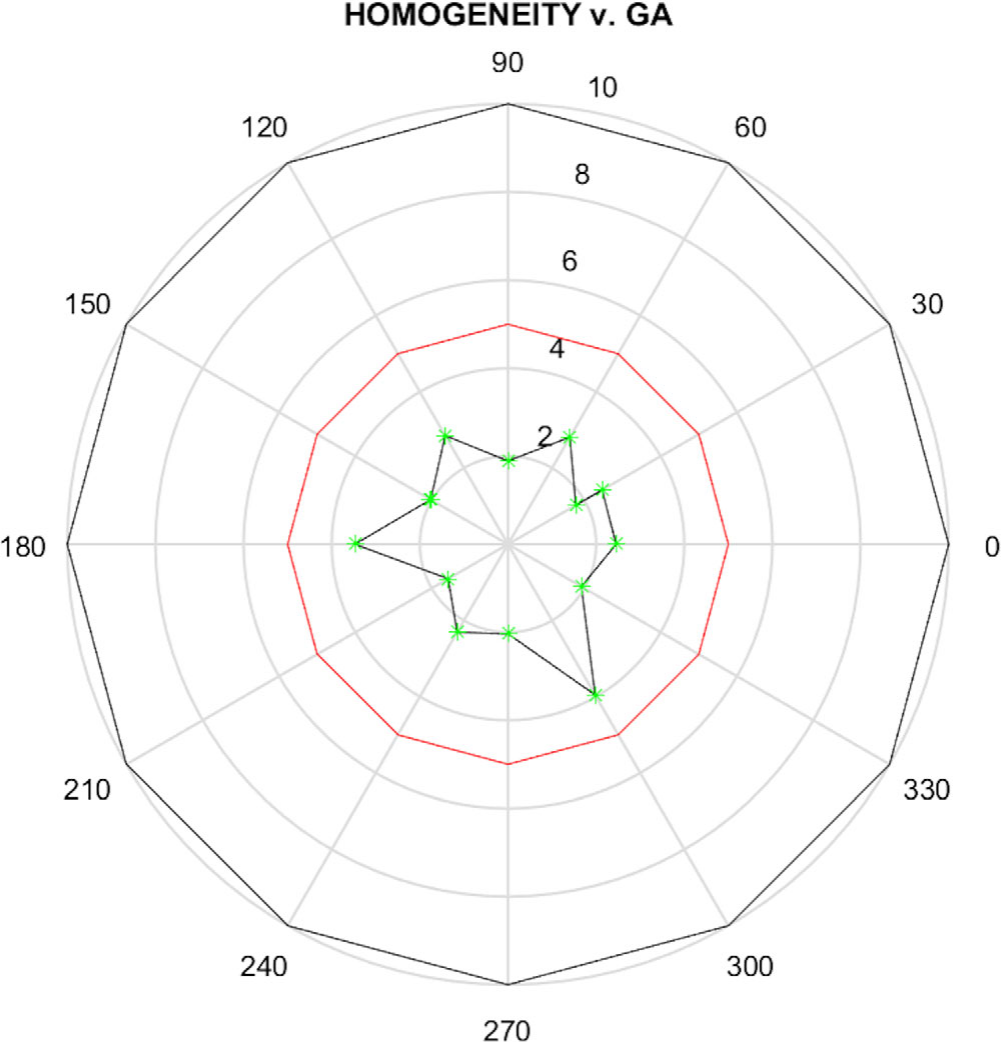
Radar plot of magnetic field homogeneity values (green stars, in ppm) at discrete gantry angles after shimming. The concentric gray circles are labeled 2, 4, 6, and 8 ppm, while the red circle marks the 5 ppm tolerance. All measured values remain below 5 ppm.

**FIGURE 8:**
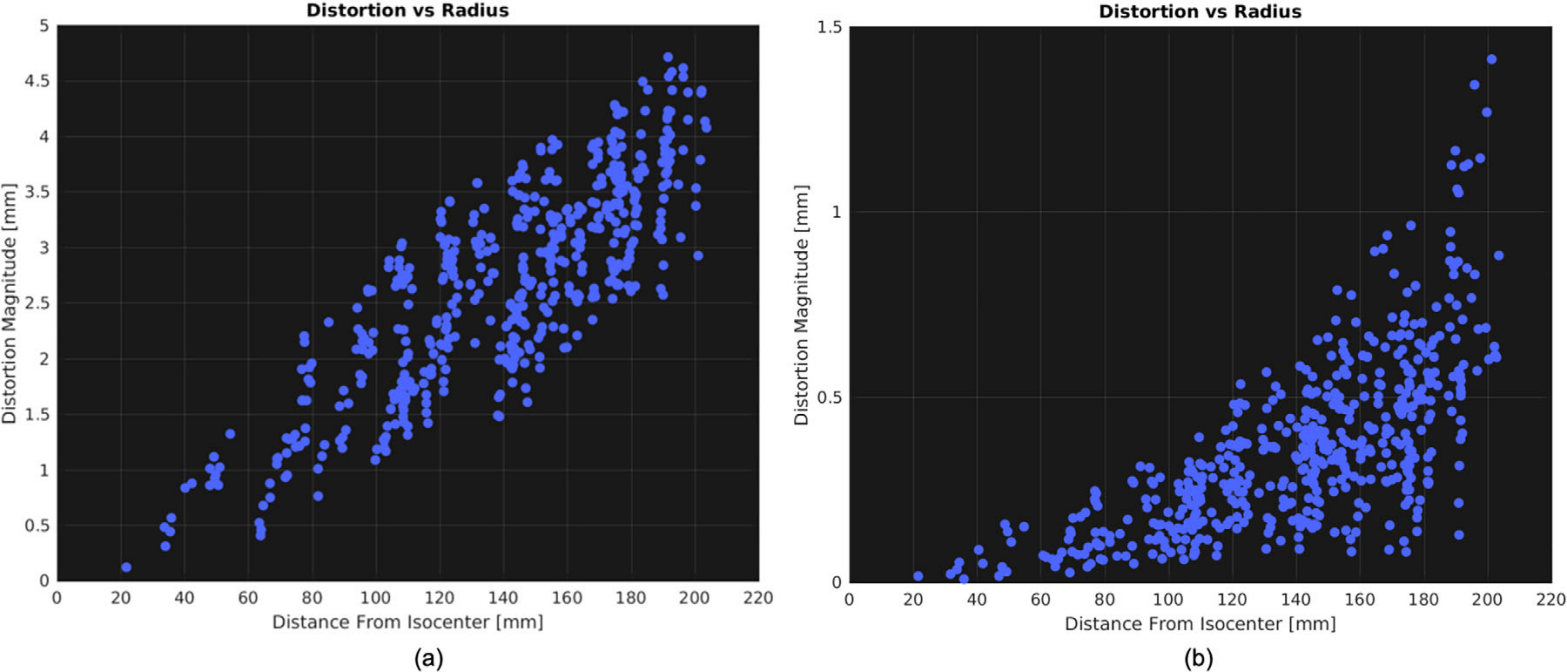
Effect of out‐of‐tolerance magnetic field homogeneity on TRUFI clinical‐sequence images of a MagPhan MR image‐quality phantom. The vertical axis indicates the measured distortion (mm), and the horizontal axis shows the radial distance from isocenter (mm), extending to 200 mm. Each blue point represents a measured distortion value. In (a), before shimming, the mean distortion is 2.420 mm. In (b), after shimming, the mean distortion is reduced to 0.280 mm, illustrating the significant improvement in MR image quality.

We also verified the MRI component, specifically magnet field homogeneity (<  5 ppm within a 24 cm DSV).^41^ Figure 5 shows an out‑of‑tolerance homogeneity value (5.7 ppm) at 60°, which was corrected through shimming. Figure 7 displays a radar plot of magnetic field homogeneity for various gantry angles after shimming, demonstrating that all values now fall below 5 ppm. To quantify the impact of re‑shimming on geometric distortion, we used a commercial large phantom (MagPhan, The Phantom Lab, USA). Figure 8 illustrates how image distortion improved from a mean of 2.420 mm before shimming to 0.280 mm afterward, underscoring the importance of maintaining magnet field homogeneity below 5 ppm.

Although the manufacturer‐supplied phantoms and protocols meet the basic requirements for acceptance testing, we recommend more comprehensive phantoms for establishing rigorous long‐term QA baselines. For instance, the 2D VR spatial integrity phantom is smaller than the 0.35T MR‐Linac bore, limiting assessment of potential larger‐scale geometric distortions. Likewise, the ACR body phantom used in 2D scanning mode is modest in size for this system. Larger, commercially available phantoms are preferable for more extensive distortion evaluations and for setting QA baselines.^41^, ^42^

Beam scanning with an MR‐compatible water tank is recommended for detailed characterization of beam profiles, percentage depth dose (PDD), and FOFs during acceptance and commissioning. For beam‐quality testing, 0.35 T MR‐Linac vendors typically evaluate the tissue–maximum ratio (TMR) at 10 cm depth using a TG‐51 water tank. Measurements acquired at dmax (1.5 cm) and 10 cm for the reference field are compared with Monte Carlo–based TPS calculations; in our case, the measured and calculated TMR values were 0.7512 and 0.75387, respectively. Although 2D arrays and 1D water tanks can be used,^43^ they often lack the accuracy and flexibility needed for small fields. We therefore performed beam scanning with an MR‐compatible water tank, measured selected FSs for PDD and profiles, and visually compared the results with TPS data to verify agreement (data not shown). Further research is needed to establish standardized guidelines for beam characterization—focusing on PDD and profile measurements—to quantify magnetic‐field effects on the concordance between measurements and TPS calculations.

## 5 CONCLUSION

The acceptance test is a critical step to verify the validity of the newly purchased Linac and to establish the subsequent QA baseline. The procedures for the 0.35T MR‑Linac were categorized into three components: mechanical, dosimetric, and MRI, with phantoms and devices chosen according to the manufacturer's requirements and specifications. Overall, the acceptance criteria were met except for very small FSs and couch beam attenuation at certain gantry angles. This manuscript provides a comprehensive account of the acceptance testing process and the devices used in a single institution's experience.

## AUTHOR CONTRIBUTIONS

**Mateb Al Khalifa**: Conceptualization, Acquisition, Interpretation of data, Methodology, Validation, Drafting the work, Revising the draft, Final approval of the version. **Tianjun Ma**: Revising the draft, Final approval of the version. **Haya Aljuaid**: Revising the draft, Final approval of the version. **Siyong Kim**: Revising the draft, Final approval of the version. **William Y. Song**: Revising the draft, Final approval of the version.

## CONFLICT OF INTEREST STATEMENT

The authors declare no conflicts of interest.

## Supporting information



Supporting Information
