# Supplementary material for: Acceptance testing of a 0.35 T MR‐Linac: procedures, QA baseline, and system limitations
Source: J Appl Clin Med Phys. 2026 Feb 14;27(2):e70488. doi: 10.1002/acm2.70488 (PMC12906295; doi:10.1002/acm2.70488)
Supplement: Supplementary file 1 — Supporting Information [file ACM2-27-e70488-s001.docx]

Table 10 Overview of the mechanical, dosimetric, and MRI verification tests. Each row summarizes the test description, acceptance criteria, applicable guidance, the phantoms/devices used, and any general remarks.

| Test Category | Description | Criteria | Guideline | Phantom/Device | Comment |
| --- | --- | --- | --- | --- | --- |
| Mechanical | Coincidence of radiation, MRI, and laser isocenters | Coordinates coincide within a 1 mm radius | AAPM TG-142 | VR QA phantom and EBT3 film | The radiation and MRI isocenters share the same location (treatment isocenter), whereas the laser isocenter is positioned 155 cm away (virtual isocenter). |
|  | MLC radiation field size accuracy | Measurement vs. TPS MC: ≤ ±2 mm | Manufacturer specification | EBT3 film for fields ≤ 1.6 cm²; ICP for fields ≥ 4.15 cm² | Evaluated field sizes: 0.83, 1.66, 4.15, 9.96, 14.94, 19.92, 24.07 cm², and 27.2 × 24.07 cm². Each field size was determined by the FWHM in the X/Y beam profiles. |
|  | MLC transmission and inter‐leaf leakage | Maximum leakage < 1 % (over a 1 × 1 cm² area) | IEC 60601‐2‐1 (201.10.1.2.103.2.1 X‐RADIATION) | EBT3 film | The average MLC leakage, measured over a 30 × 30 cm² region of interest (ROI), remains below 0.375 % relative to a 9.96 × 9.96 cm² reference field at isocenter. |
|  | MLC linearity and position accuracy | ≤ ±2 mm | Manufacturer specification | EBT3 film | MLC beam centers are aligned within 2 mm of their nominal positions. |
| Dosimetric validation | Field profile shape and symmetry | Measurement vs. TPS MC; symmetry ≤ ±2 % | Manufacturer specification | ICP | Field sizes of 9.96 × 9.96 cm² and 27.2 × 24.07 cm² were measured at gantry angles of 0°, 90°, and 270°. Off‐axis ratios were evaluated at two points equidistant from the CAX. |
|  | Field penumbra | Measurement vs. TPS MC; 20%–80% penumbra ≤ 1 mm | Manufacturer specification | EDGE detector in a water tank for fields ≤ 1.6 cm × 1.6 cm; ICP for fields ≥ 4.15 cm × 4.15 cm | Field sizes of 0.83, 1.66, 4.15, 9.96, 14.94, 19.92, 24.07 cm², and 27.2 × 24.07 cm² were assessed at a 5 cm depth. |
|  | Couch attenuation | Measurement vs. TPS MC: ±3 % | Manufacturer specification | VR daily QA phantom, Ion chamber (A28) | A 9.96 × 9.96 cm² field was measured at gantry angles 140°, 160°, 180°, 200°, and 220°, with 90° and 270° averaged as the reference. |
|  | Reference dosimetry | ± 1% | AAPM TG‑51 addendum | MRI‐compatible 1D water tank and PTW farmer‐type ionization chamber (TN30013) | Reference conditions include 1 cGy/MU at d_max_, a 9.96 × 9.96 cm² field, an SAD of 90 cm, an SSD of 80 cm, and a depth of 10 cm. |
|  | Field output factor | Not specified (minimum recommended) | TRS 483 | THALES 3D MR Scanner water tank, Exradin^®^ W2 PSD | Measurements were performed at an SSD of 85 cm and a detector depth of 5 cm. |
|  |  |  |  |  |  |
|  | Dose and latency gating | Dose delivery to a moving target vs. stationary target: ±2 % | Manufacturer specification | CIRS phantom, Ion chamber (A28) | A waveform motion pattern was configured with a 12 mm amplitude and a 4 second period. |
| MRI verification | Magnet field homogeneity | <5 ppm within a 24 cm DSV | Manufacturer specification | 24 cm spherical phantom | Measurements were performed at multiple gantry angles (every 30°). |
|  | Spatial integrity | < 2 mm over a 35 cm FOV (90% of points) and < 1 mm over a 20 cm FOV (100% of points) | Manufacturer specification | 2D VR Spatial Integrity phantom | Measurements were acquired in the axial, coronal, and sagittal orientations at the CAX. Off‐axis positions of ±7 cm and ±12.5 cm were included to assess the full imaging FOV. |
|  | SNR | Torso and head/neck coils: > 30 for sagittal and transverse, > 25 for coronal; Body coil: ≥ 12 | Manufacturer specification | 24 cm spherical phantom | Each coil was tested for SNR. |
|  | Uniformity | Torso and head/neck coils: > 50%; Body coil: ≥ 60% | Manufacturer specification | 24 cm spherical phantom | Each coil was tested for uniformity. |
|  | ACR | Slice position ≤ 5 mm; slice thickness 5.0 mm ± 0.7 mm; high contrast ≥ 0.9 mm; low contrast > 18; percentage ghost < 0.025; uniformity > 87.5 % | Manufacturer specification | ACR body phantom | 2D T1‐ and T2‐weighted scans were performed. The TRUFI sequence was not included. |
